# Impact of healthy diet and physical activity on metabolic health in men and women: Study Protocol Clinical Trial (SPIRIT Compliant)

**DOI:** 10.1097/MD.0000000000019584

**Published:** 2020-04-17

**Authors:** Oscar Bergens, Jort Veen, Diego Montiel-Rojas, Peter Edholm, Fawzi Kadi, Andreas Nilsson

**Affiliations:** School of Health Sciences, Örebro University, Örebro, Sweden.

**Keywords:** aging, body composition, exercise, healthy diet, metabolic syndrome, systemic inflammation

## Abstract

**Introduction:**

Healthy dietary patterns and physical activity (PA) represent important lifestyle behaviors with considerable potential to influence on age-related metabolic health. Yet, data on the combined effects of these lifestyle behaviors on metabolic health including low-grade systemic inflammation in aging populations remain scarce. Therefore, this protocol describes a randomized controlled trial aiming to examine the impacts of healthy dietary patterns alone or combined with PA on metabolic health in middle-aged and older men and women.

**Material and Methods:**

The ORUDIET study is a 3-arm randomized controlled 16-week trial: Healthy Diet (HD), Healthy diet plus PA (HD-PA), and control (CON). The trial is open label, randomized with allocation concealment, parallel groups with passive controls. Participants without overt disease aged between 55 and 70 years, with BMI below 35, a current intake of a maximum of 1 serving of fruit and vegetable per day, and noncompliance to PA guidelines are eligible for inclusion. Participants in HD are instructed to increase fruit and vegetable intake to 5 servings per day (equivalent to 500 g). Participants in HD-PA receive the same dietary intervention as the HD and are additionally instructed to engage in moderate-to-vigorous physical activities for at least 150 minutes per week. The primary study outcomes are changes in metabolic and inflammatory health biomarkers. Secondary outcomes are changes in body composition and perceived health.

**Ethics and dissemination:**

The study protocol has been approved by the ethical review board in Uppsala, Sweden. The results will be published in peer-reviewed journals and disseminated in national and international conferences.

**Trial registration number:**

NCT04062682 Pre-results

## 1 Introduction

Population aging is a worldwide trend and is becoming a growing public health challenge. Indeed, aging is associated among others with a gradual decline in physical function leading to an increase in care-dependent older adults with related increases in health care costs.^[1]^ A slight and chronic elevation in circulating biomarkers of systemic inflammation has typically been reported in older adults.^[2]^ This age-related low-grade systemic inflammation is suggested to precede the onset and development of several chronic diseases, including diabetes and cardiovascular diseases.^[2]^ Together with systemic inflammation, abdominal obesity, hypertension, dyslipidemia and hyperglycemia, collectively defined as the metabolic syndrome (MetS), represent a cluster of strong risk factors for the development of chronic diseases.^[3]^

It is postulated that dietary habits are important lifestyle behaviors with a potential to readily impact on obesity and MetS, factors that have a strong inflammatory pathogenesis contributing to a state of metabolic inflammation (i.e., “metaflammation”).^[2]^ Healthy dietary patterns characterized by high intakes of fruit and vegetable, whole-grain products, low fat dairy, nuts and seeds, and limited intakes of saturated fats, sodium, and red meat have been recommended by global health organizations.^[4]^ Unfortunately, global data show that intakes of healthy food items, with fruit and vegetables in particular, are well below recommended amounts^[5,6]^ and approximately 4 million deaths globally have been attributed to poor dietary habits.^[7]^

Recent observational studies have indicated favorable influences of healthy diets characterized by higher fruit and vegetable intakes on several biomarkers of inflammation, including C-reactive protein (CRP), interleukin-6 (IL-6), IL-10, and tumor necrosis factor-α (TNF-α),^[8–10]^ and components of MetS.^[11–13]^ However, studies based on experimental settings exploring the effects of healthy diets characterized by increased fruit and vegetables on markers of inflammation^[14–16]^ and metabolic risk^[17,18]^ have yielded inconclusive results. For example, although beneficial effects on body composition and insulin levels have been reported in middle-aged overweight and obese individuals,^[17]^ others have failed to show corresponding effects in middle-aged adults with MetS.^[18]^ Furthermore, healthy dietary patterns characterized by increased fruit and vegetable have been linked to reductions in levels of pro-inflammatory biomarkers, including plasma CRP, in some^[15,16]^ but not all studies.^[14]^ Notably, data exploring how the adoption of healthy dietary patterns influences on inflammatory and metabolic biomarkers during the aging process, specifically, are scarce. This paucity of data is unfortunate given the increased prevalence of MetS^[19]^ together with elevations in circulating inflammatory biomarkers with advancing age^[2]^ making the growing population of older adults an important target for preventive health efforts.

Moreover, given the well-established health benefits from adhering to a physically active lifestyle, where an amount of 150 weekly minutes in moderate-to-vigorous PA (MVPA) together with regular engagement in strengthening activities are advocated,^[20]^ lifestyle promotion of healthy aging may not be limited to promotion of healthy diet only, but rather in combination with promotion of increased PA levels. However, surprisingly few randomized controlled trials (RCTs) have previously explored whether a combination of a healthy diet characterized by increased fruit and vegetable intakes together with increased PA levels may elicit greater health benefits compared to changes in diet only.^[21]^

Taken together, new knowledge regarding impact of healthy dietary habits alone or combination with PA on cardiometabolic and inflammatory outcomes is warranted, as it can fuel preventive actions aiming to combat age-related inflammatory and metabolic abnormalities in the growing aging population. Therefore, the ORUDIET study protocol outlines a 3-arm RCT designed to determine the impact of healthy dietary pattern alone or in combination with PA on health outcomes in a population of men and women

## 2 Methods

### 2.1 Study design

**Figure 1:**
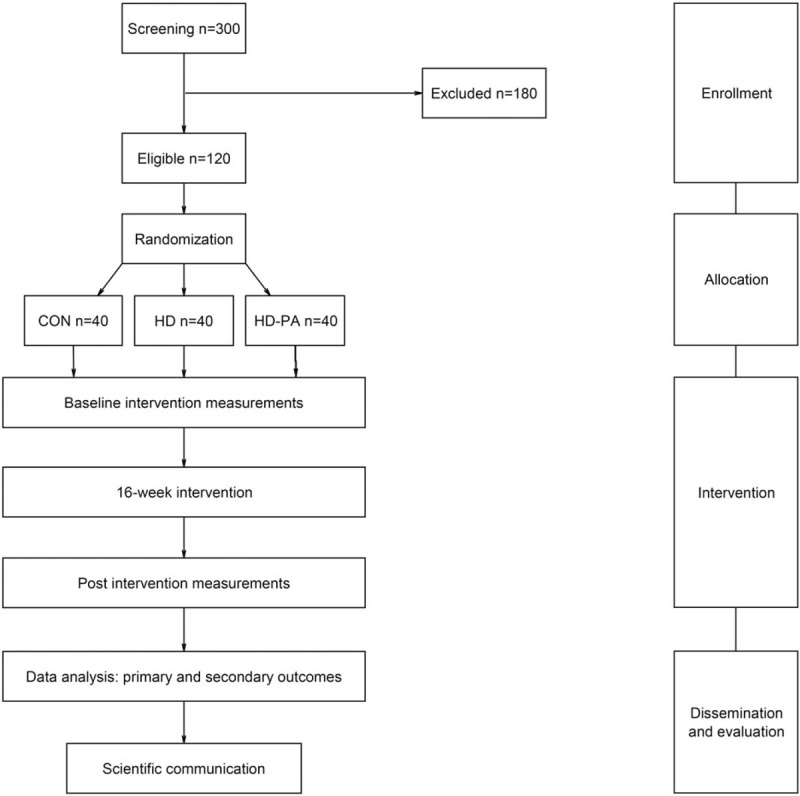
CONSORT flow diagram of the ORUDIET trial.

The ORUDIET is a 3-arm RCT (start date: February 2020). The trial is open label, randomized with allocation concealment, parallel groups with passive controls and includes a healthy diet group (HD), a healthy diet plus PA group (HD-PA), and control group (CON). This research protocol was designed without patient involvement. Participants were not invited to comment on the study design. ORUDIET trial is registered at www.clinicaltrials.gov as NCT04062682. A CONSORT flow diagram is shown in Fig. 1.

### 2.2 Study population and group allocation

A sample of 300 adults (150 men and 150 women; age 55–70 years) are being recruited through local advertisement and screened for inclusion. On the basis of the screened sample, 60 men and 60 women are anticipated to be randomized into 1 of the 3 groups (20 men and 20 women per group). Randomization will be conducted by an independent statistician using computer-generated random numbers. Stratification by biological sex will be conducted. On the basis of data from previous studies, clinically relevant effects on cardiometabolic outcomes can be detected with 20 participants of the same sex per intervention arm, with a statistical power ≥0.8 and an alpha-level set to *P* < .05.

The inclusion criteria for the study are age between 55 and 70 years, current intake of a maximum of 1 serving of fruit and vegetable per day, and noncompliance to physical activity guidelines (less than 150 weekly minutes of MVPA and less than 2 sessions of strengthening activities). The exclusion criteria are body mass index (BMI) >35, food allergies, movement disability, presence of cardiovascular, metabolic, rheumatologic or psychiatric diseases. Participants will attend a medical screening to confirm eligibility for the study entry. During screening, they will provide information on sociodemographics, medical history, and dietary and physical activity behaviors. Randomization is performed by block-design to ensure equal numbers of participants assigned into the 3 groups stratified by sex.

### 2.3 Intervention

Participants in HD and HD-PA groups are instructed to increase fruit and vegetable intake to 5 servings per day (equivalent to 500 g) during 16 weeks. Several menus are composed by a nutritionist to achieve guidelines for healthy dietary habits. Participants attend 2 group-based dietary counselling sessions during the start of the 16-week intervention period. During these sessions, participants are helped to identify potential barriers and facilitators for implementing new dietary habits in order to support adherence during the intervention period. In addition, adherence to the healthy diet is reinforced using a behavioral support package (reading materials on guidelines, recipes, and cooking tips on healthy food choices) provided through a digital platform during the intervention period. Participants in HD are instructed to keep their habitual physical activity level.

Participants in HD-PA receive the same dietary intervention as the HD and are additionally instructed to engage in physical activities corresponding to at least 150 weekly minutes of moderate-vigorous intensity according to current guidelines. For this purpose, participants receive a compendium of physical activities all corresponding to a at least moderate intensity. Together with trained staff from Örebro University, participants are helped to choose appropriate activities to perform during the intervention period. The 2 counselling sessions before the intervention also include identification of potential barriers and facilitators for being physically active. A digital platform with behavioral support on adherence to PA guidelines (reading materials on guidelines, tips on activities, equipment, and safety issues) is provided alongside support for healthy diets. Participants in the control group are instructed to maintain their habitual dietary and physical activity habits.

### 2.4 Primary and secondary outcomes

The primary outcomes are the changes in metabolic and inflammatory health biomarkers (see biomarkers description below). Secondary outcomes are changes in body composition parameters and perceived health as outlined below.

### 2.5 Procedure and data collection

Recruited participants attend a first visit at the laboratory in order to screen for eligibility criteria. Assessments of physical function and medical screening including measurement of blood pressure are performed and dietary intake including fruit and vegetable intake is determined by a food-frequency-questionnaire (FFQ). Participants are instructed to wear an accelerometer for 1 week to assess habitual PA level. Eligible participants attend a second visit for assessment of body composition and blood sample based biomarkers. Questionnaires on facilitators and barriers for a healthy lifestyle and health-related quality of life are administered.

At the start of the 16-week intervention period, participants randomized to HD and HD-PA attend the counselling sessions held by trained personal in promotion of healthy lifestyles. During these sessions, experiences regarding facilitators and barriers for behavioral change are shared between participants. Information on adherence to HD and HD-PA is self-reported bi-weekly using a digital platform. At the end of the 16-week intervention, measurements of physical function, body composition, dietary intake, PA level, and analysis of blood-based biomarkers are repeated.

### 2.6 Measures

#### 2.6.1 Assessment of dietary patterns

Dietary data are collected using a validated 84-item food-frequency questionnaire (FFQ).^[22]^ Intake frequency has 9 fixed alternatives (never, occasionally, 1–3 times/month, 1 time/week, 2–3 times/week, 4–6 times/week, 1 time/day, 2–3 times/day, ≥4 times/day).

#### 2.6.2 Assessment of PA

Habitual PA will be assessed by the Actigraph GT3x (Actigraph, Pensacola, FL) activity monitor during a week. Count cut-points for sedentary time (<100 counts per min), time in light-intensity PA (LPA) (100–2019 counts per min), and moderate-to-vigorous intensity PA (MVPA) (>2019 counts per min) will be applied as previously described.^[23]^ Adherence to PA guidelines is assessed bi-weekly by self-report.

#### 2.6.3 Anthropometrical assessments

Body weight and height will be measured using standardized equipment and procedures. Waist circumference is measured to the nearest 0.1 cm with a steel tape at the midpoint between iliac crest and lower costal margin. A bioelectrical impedance measure is performed using the Tanita MC-780 Multi-Frequency segmental body composition analyzer (Tanita, Amsterdam, The Netherlands) allowing to determine total as well as segmental fat mass and fat-free mass for the trunk, left and right arms, and legs. All anthropometric measurements are performed after an overnight fast and participants are asked to avoid alcohol and not to engage in any strenuous physical activity 24 hours before the measurement.

#### 2.6.4 Assessment of blood pressure

Systolic and diastolic blood pressures are measured manually after a 15-minute rest in the supine position using a mercury sphygmomanometer.

#### 2.6.5 Assessment of physical function

A squat jump test is performed on a force platform (Kistler 9281 B; Kistler Nordic AB, Sweden), where the participant starts from a static position with knee joints bent at a 90° angle. The hands are kept on the hip during the jump. Participants perform 3 maximal trials separated by 1.5 minutes of rest. The highest recorded maximal ground reaction force from the 3 trials is calculated from the concentric phase of the squat jump. Handgrip strength is assessed by standardized procedures using Jamar handheld dynamometer (Patterson Medical, Warrenville, IL). A 6-minute walk test is performed by walking a corridor of 30 m in length during 6 minutes, and the total distance completed is recorded. A 5 sit-to-stand test is performed where participants are instructed to stand fully upright from a chair and sit down and repeat this sequence 5 times.

#### 2.6.6 Blood sampling

Blood samples are obtained after an overnight fast and blood is collected by venipuncture from an antecubital vein, centrifuged at 4000 rpm for 10 minutes and stored in −80°C.

#### 2.6.7 Assessment of metabolic health biomarkers

The clinical metabolic markers fasting glucose and insulin, glycated hemoglobin, total cholesterol, high-density lipoprotein cholesterol (HDL-cholesterol), triglycerides, aspartate aminotransferase, and alanine aminotransferase are assessed. Prevalence of metabolic syndrome will be determined according to the criteria set by the International Diabetes Federation: waist circumference above ≥94 cm for men and ≥80 cm for women, along with any of the following; raised triglycerides, ≥1.7 mmol/L or specific treatment for lipid abnormalities; reduced HDL-cholesterol, <1.03 mmol/L or <1.29 mmol/L or specific treatment for lipid abnormalities; raised blood pressure, ≥130 mm Hg systolic or ≥85 mm Hg diastolic or treatment of previously diagnosed hypertension; raised fasting plasma glucose, ≥5.6 mmol/L or previously diagnosed diabetes type II.^[3]^

The inflammatory biomarkers IL-6, IL-10, IL-18, fibrinogen, adiponectin, leptin, 12 are assessed using commercially available ELISA kits (Mercodia, Uppsala, Sweden) and high-sensitivity CRP (hs-CRP) is measured using a fully automated immunoturbidimetric assay (Advia 1800; Chemistry System, Siemens, Germany).

For the analyses of molecular lipids (lipidomics), ultra-high performance liquid chromatography combined with quadrupole-time-of-flight mass spectrometry (UHPLC-QTOFMS) will be utilized as previously described.^[24]^ The method is semiquantitative, based on normalization and quantitation of lipid-class specific compounds. In brief, total lipid extraction from serum samples is performed using a chloroform:methanol mixture after addition of internal standards. External standard is added after the extraction. Lipid extracts are then analyzed on the Q-Tof Premier mass spectrometer: Agilent Technologies (Santa Clara, California) with the Acquity ultra performance liquid chromatography BEH C18: Waters (Milford, Massachusetts) 2.1 × 100 mm column with 1.7 μm particles. The data are collected at a mass range of m/z 300 to 1200 with 0.2 s scan duration in ESI+ mode and are processed using the MZmine 2 software: open source software (available at: http://mine.github.io). Lipids are identified using an internal spectral library or with tandemMS, and data are normalized using the internal standards, representative of each class of lipids present in the samples, as previously described.^[24]^

#### 2.6.8 Assessment of facilitators and barriers for change in diet and PA behaviors

Questionnaires on perceived barriers for healthy eating^[25]^ and for being physically active^[26]^ are being used to explore barriers for adopting healthier lifestyles. Moreover, semi-structured interviews will be conducted on a total of 20 men and 20 women. The interviews will be partly based on previously published protocols developed to unveil perceptions about age-related changes in diet and PA behaviors.^[27]^

#### 2.6.9 Assessment of health-related quality of life

Self-perceived physical and mental component summary scores will be derived from the 36-Item Short-Form Health Survey (SF-36).

### 2.7 Data analysis

Data analysis is performed according to the intention-to-treat principle. Mixed-models analysis of variance including within-subject and between-subject factors and related interaction effects are conducted. Moreover, advanced bioinformatics and multivariate statistical approaches (principal component analysis, multinomial logistic regression, neural networks, Bayesian networks, grade-of-membership analysis) will be applied for the analysis of lipidomic data. Results from questionnaires will be summarized based on quantitative measures of frequencies regarding identification of common perceptions of motives, facilitators, and perceived barriers for change in diet and PA behaviors. Material from interviews will be transcribed and analyzed using qualitative content analysis, with the aim of finding the key themes that can enhance understanding of the conditions for lifestyle change. As the interview protocol will be partly based on a previously published theoretical framework, a framework analysis will also be conducted, which involves coding data according to their importance rather than simply frequency following a systematic 5-step procedure.

### 2.8 Quality assurance/monitoring

Trained research staff will be responsible for entry of screening, enrollment, and medical records into the study. Standard of procedures (SOPs) for blood sampling, including sample preparation and biological analysis, blood pressure, and body composition measurements are established according to highest scientific standards. A data monitoring committee (data manager and statistician) will ensure accurate and efficient data collection and analysis; confidentiality and, on-demand study monitoring reports. Only principal investigator has access to the final trial data set. No sponsors or competing interests are identified. The trial monitoring committee, comprising senior physicians at the clinical facility, which are independent of the principal investigators, performs monitoring of the intervention by monthly in-person meetings with primary care providers and experienced clinicians.

### 2.9 Ethics and dissemination

The study protocol has been approved by the regional ethical review board of Uppsala, Sweden (Dnr 2019–04434S). All research activities are performed within the frame of fundamental ethics principles, including those set out in the declaration of Helsinki. The study protocol strictly follows current regulations regarding informed consent, confidentiality, and anonymity. Informed consent document including explanation and purpose of the project, description of procedures, potential discomfort, and benefits are provided. Participants are also informed that participation is voluntary and withdrawal possible at any time. Collection of blood samples is the only invasive procedure in the study. Predefined adverse events are reported to the trial monitoring committee, which ensures participants’ safety. The intervention is based on nonpharmacological approach (implementation of healthy food). However, any food allergy is checked before inclusion. All tests are conducted by certified personnel. In case of abnormal test readings and discovery of health-threatening conditions, participants will be referred to further medical examinations. All personal data files will be stored on a password-protected server only accessed by authorized members of the research team and data are handled according to the Personal Data Act. A data sharing plan has been established, where de-identified individual data on participant characteristics and main study outcomes will be made available on reasonable request once the results are published. This RCT is registered in clinicaltrials.gov (NCT04062682 Pre-results).

## 3 Discussion

A novel feature of the present study protocol lies in its 3-armed approach allowing to examine to what extent healthy diets alone or in combination with physical activity improves metabolic health in aging populations. Exploration of a large panel of advanced biomarkers of metabolic health encompassing systemic inflammation and molecular lipids signatures would provide new insights into mechanisms behind lifestyle-related impacts on metabolic health.

Healthy dietary patterns characterized by high intakes of fruit and vegetables may infer protective health effects through various mechanisms including increased anti-oxidative defences^[28,29]^ and reduced circulating levels of several inflammatory biomarkers.^[15,16]^ It is currently proposed that an interaction between a systemic pro-inflammatory environment and the metabolic machinery causes a state of metabolic inflammation (metaflammation), which promotes chronic disease progression.^[2]^ Thus, our study protocol provides an in-depth evaluation of the adequacy of current guidelines for healthy diets in general and fruit and vegetable intakes in particular to combat age-related metabolic deteriorations. Furthermore, our study protocol provides new information on the additive metabolic impact of PA alongside adherence to guidelines for healthy diet in aging populations without manifest disease.

In conclusion, given the global epidemic of metabolic diseases, healthy lifestyles in late adulthood hold important potential to counteract this trend. There is an urgent unmet need of knowledge about the benefits of combining healthy diet and PA lifestyles derived from randomized controlled settings. In this way, this study protocol responds to a societal need, by covering behavioral factors of importance for successful implementation of healthy dietary and PA habits in a community setting.

## Acknowledgment

This work has been designed within the frame of the Food and Health Initiative at Örebro University. We thank the European Commission through the Marie Skłodowska-Curie Actions, Co-funding of Regional, National and International Programmes (MSCA COFUND) for supporting one author (JV).

## Author contributions

**AN, FK:** initiation of the study design, conducting primary statistical analysis, writing of manuscript, approval of final draft

**OB, JV:** refinement of the study protocol, writing of manuscript, approval of draft

**PE, DMR:** refinement of the study protocol, approval of final draft

**DMR, PE:** revision of manuscript
